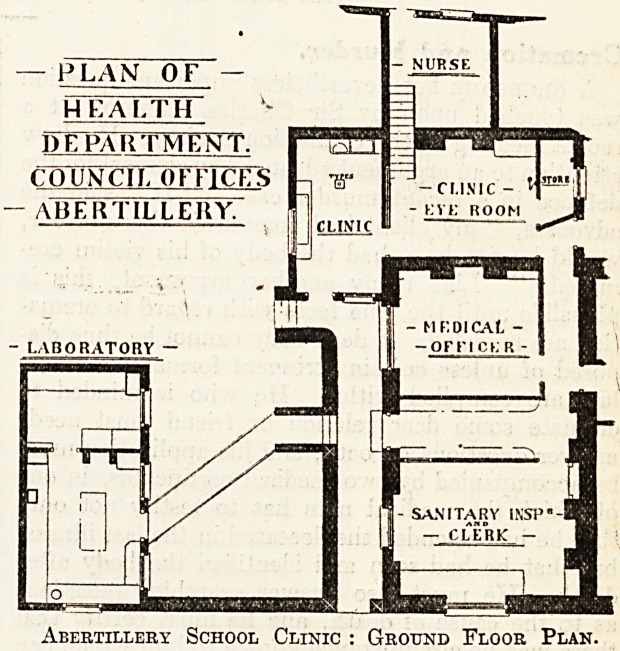# The Abertillery School Clinic

**Published:** 1912-04-13

**Authors:** 


### THE ABERTILLERY SCHOOL CLINIC.

### Its Buildings, Organisation, and Advantages.

We give the description of this clinic as an example of what has already been done. It is by no means an ideal clinic, as the ground plan attached will show, but it presents some interesting features, for an account of which we are indebted to Dr. Weaver's report to the local education committee.

The clinic is one of the "adapted type" models —that is to say, it is simply a portion of an ordinary dwelling house which has been converted into a centre of treatment. The house was formerly used by the town surveyor and has now been taken over by the health department.

At the extreme south end is the sanitary inspector's office. Between this and the medical officer's room is a lobby-hall fitted with seats and used as a waiting-room. Corridors at both ends connect this lobby with the examination rooms, of which there are two. At the back of the examination rooms is a special sitting-room for the use of the nurse in attendance. In connection with the main building is a laboratory for bacteriological and public health work.

The examination rooms consist of two connected rooms, with a small storage-room at the back. The large room is fairly light and airy, and is used both for examining and for treating children. It possesses a double sink, with adequate water-supply, a water heater, steriliser, instrument cabinet, and cupboard accommodation for instruments, drugs, dressings, and other necessary materials. The furnishing is plain and aseptic, and the frontispiece to Dr. Weaver's report gives a good general view of the room. The middle room, which is dark, is comparatively small and is used as a room for eye work. "It is fitted with Mackenzie parallel bracket gas-lamp, set of trial lenses, etc., for eye testing. As the room was shorter than six metres, it was found necessary to place the test types in the other clinic room, and to make a window for viewing them in the glass partition between the rooms."

The clinic is open three times a week during the sessions, on Tuesday and Thursday afternoons and on Saturday mornings. The cases seen are all referred to the clinic for treatment or further examination by the school doctors or teachers. Dr. Weaver examines the children and prescribes the treatment, which in the majority of cases is carried out by the school nurse. During the first half of the winter session of 1910, 228 cases were seen, with 671 visits. The defects noted were as follows: Defective eyesight, squint, external eye disease, deafness, otorrhœa, scabies, ringworm, sores on scalp, other skin diseases. The examination of the eyes appears to be very thorough. A mydriatic is used in every case. Arrangements have been made with local opticians to supply spectacles according to the clinic's prescriptions at the following rates: Steel frames, spherical lenses, 1s. 9d. per pair; plano-cylindrical lenses, 2s. 3d. per pair; sphero-cylindrical lenses, 3s. per pair.

The clinic is under the control of a special committee, and a charge is made for treatment but not for examination only. Refraction estimations are considered as examinations and are not charged for. During the first month the charge is at the rate of 1s. per month; if after that time the child still requires treatment, the charge is 6d. per month. Where necessary, owing to the poverty of the parents, free treatment is given, inquiry being made into all such cases in order to verify the parents' statements. Operations for the removal of adenoids and tonsils are not performed at this clinic.

Dr. Weaver, in concluding his report, gives an interesting review of the working of the clinic. The

Abertillery School Clinic: Ground Floor Plan.

annual cost of the undertaking has been very small, and the initial outlay (£110) was largely due to the expenses incurred in altering the rooms and, of course, in providing apparatus. It is not stated if an $x$-ray installation is provided at the clinic; the photograph at least does not show such a necessary adjunct. The following are among the advantages Dr. Weaver claims for the clinic:—

Means are available for the more complete examination of children, who cannot be adequately examined during routine medical inspection at the schools.

Every defective child is assured of having an opportunity of receiving treatment (so far as scope of the clinic permits), in spite of the poor circumstances or apathy of its parents.

Children obtain prompter treatment, and therefore return to school and earn grants sooner than formerly. Others, particularly those with defects of eyesight and hearing, are enabled to profit better by their school instruction.

The clinic co-ordinates the work of medical inspection with that of treatment, and so ensures that the money expended on the former is not wasted.

Private medical practitioners gain rather than lose, for opportunity is given to procure private treatment. Parents, being urged to obtain treatment, in many instances send their children to private practitioners. Some of the conditions treated in the clinic are not readily dealt with by local, private, or colliery doctors, either on account of the tediousness of the treatment, or the absence of the necessary facilities, and of the services of a trained nurse.

The "responsibility of parents" is increased, because, all excuse for neglect of obtaining treatment for their children having been removed, parents will be compelled to do their duty to their children's curable defects.

The bogey of "diminished parental responsibility" can be completely dismissed from all matters of school medical inspection. No Act yet placed on the Statute Book has so justified its existence, or done so much to increase the responsibility of parents as regards the cleanliness and health of their children as the Education (Administrative Provisions) Act. The charges levelled against the Act have arisen from a false conception of its aims and methods, and anyone acquainted with the *efficient* administration of medical inspection knows full well that it has led to a marked improvement in the physical condition of the children being *effected by the parents*, and to hundreds of prosecutions of negligent parents being instituted, while the cry of the hospitals and dispensaries that they cannot deal with the influx of cases caused by medical inspection is proof positive that a revolution in the treatment of children's defects has occurred.

I am glad also to be able to record that there has been no sign of friction with the local medical practitioners, in fact cases have been referred to the clinic by them, instead of being sent to special hospitals at a distance.

## Figures and Tables

**Figure f1:**